# Current practices of waste management in teaching hospitals and presence of incinerators in densely populated areas

**DOI:** 10.1186/s12889-021-11389-1

**Published:** 2021-07-07

**Authors:** Salma Khalid, Najibul Haq, Zia-ul-Ain Sabiha, Abdul Latif, Muhammad Amjad Khan, Javaid Iqbal, Nowsher Yousaf

**Affiliations:** 1 Prime Institute of Public Health, Prime Foundation, Riphah International University, Islamabad, Pakistan; 2 Department of Medicine, Peshawar Medical College, Prime Foundation, Riphah International University, Islamabad, Pakistan; 3 Department of Community Health Sciences, Peshawar Medical College, Prime Foundation, Riphah International University, Islamabad, Pakistan; 4 Department of Environmental Sciences, University of Peshawar, Peshawar, Pakistan

**Keywords:** Current practices, Health risks, Incinerators, Teaching hospitals, Populated areas, Peshawar

## Abstract

**Background:**

Hospital waste management (HWM) practices are the core need to run a proper health care facility. This study encompasses the HWM practices in teaching hospitals of Peshawar, Pakistan and examine the enforcement of Pak HWM (2005) rules and risks through transmission of pathogens via blood fluids, air pollution during waste incineration and injuries occurring in conjunction with open burning and dumping.

**Methods:**

A questionnaire based on World Health Organization (WHO) recommendations was used to survey the selected private and public teaching hospital (*n* = 16). Site visits and personnel observations were also included in the data. It was spatio-statistically analyzed using descriptive statistics, Krushkal-wallis and Fisher’s exact tests.

**Results:**

The findings revealed that the lack of HWM practices in all surveyed hospitals (*p* > 0.05), besides statistical difference (*p* < 0.017) in waste generation/day. No proper segregation of waste from generation point to final disposal was practiced. However, the performance of private teaching hospitals (50%) was found better in terms of HWM personnel and practices. In surveyed hospitals, only nine hospitals (56.3%) were found with the incinerator facility while rest of the hospitals (43.7%) practiced open dumping. Moreover, operational parameters of the incinerators were not found satisfactory and located in densely populated areas and emitting hazardous gases.

**Conclusion:**

Proper HWM practices are not being followed in the light of WHO guidelines. Hospital waste impose serious menace to healthcare workers and to nearby population. WHO issued documents for improving HWM practices but triggered no change in Pakistan. To improve the situation, insights in this context is need for enforcement of rules.

**Supplementary Information:**

The online version contains supplementary material available at 10.1186/s12889-021-11389-1.

## Background

Globally, inadequate and improper handling of hospital’s waste is a major concern in many developing countries [1]. The effect of waste mismanagement is considerable and far-reaching in terms of serious public health consequences and has significant impacts on the environment [1–3]. Main contributing factors for increased ratio of hospital waste generation are high population growth rate, increase in number of healthcare facilities, easy access of population to the health care facilities and use of the disposable medical products [4, 5].

Worldwide, published literature on medical waste management reported poor handling, treatment and disposal of biomedical waste in many health care facilities. Hospital waste includes hazardous or risk waste and non-risk waste [1, 6]. A total of 15–20% healthcare waste is infectious, while 80–85% is non-infectious [1]. Waste produced in the hospitals either in large or small quantities carries high potential of infections and injuries [1]. The study of Almuneef et al., [7] has pointed out a strong probability that blood transmitted diseases such as AIDS, hepatitis B, hepatitis C and tuberculosis could be transferred to sanitary staff through poor handling of the hospital waste. In many low and middle income countries, hospital and municipal wastes are collected and disposed- off jointly, exposing municipal workers and public to major health risks [8–10]. According to a survey conducted by WHO (2005) in 22 developing countries including Pakistan for hospital waste management (HWM) practices, approximately 18–64% of waste disposal methods in practice were found unsuitable.

Studies in Pakistan showed that around 2 kg of waste/bed/day is produced, out of which 0.1–0.5 kg can be categorized as risk waste and their mismanagement occurs at all levels, from segregation through collection to its final disposal [11]. Despite of waste mishandling in the site of generation; its open dumping without incineration becomes the source of collection for scavengers. They collected used medical products which are recycled and re-sold in the markets [12, 13].

In many developing countries, including Pakistan, incinerators consist of primary and secondary combustion chambers for treatment of medical wastes [14], while the WHO recommended standard is a multi-chambered incinerator. In these incinerators, an absorption combination wet cyclone ensures the removal of gaseous particles, [15] whereas other low weighted air particles are preferably removed by lowering the temperature up to 200 °C [16]. In general, these incinerators work on high temperature and gas retention time frames and therefore require a huge quantity of fossil fuels to reach the desired temperature in their combustion chambers [17]. Worldwide, hospitals are setting up incineration systems based on sophisticated technology for hazardous medical wastes with lower combustion costs [18]. Although incineration is one of the final treatment option, [19] but un-regulated incineration leads to harmful effects on health including effects on sex ratio in child birth, congenital anomalies and cancer among population living nearby to an incineration plant [20, 21]. Studies on biomarkers support this: populations exposed to emissions more than others have higher biological levels of released substances [22, 23].

Likewise, several studies address various reasons, such as lack of awareness of hospital staff as well as the administration to enforce the rules, assessment of hospital waste compositions, unsafe and malpractices of hospital waste and their impacts on human health and environment [24–33], but in published literature, limited studies exist at the management status of hospital waste, current practices, and issues responsible for the gaps in the teaching hospitals of Pakistan. The present study is based to evaluate and compare the current practices of hospital waste management being undertaken by public and private teaching hospitals in Peshawar, along with structural and operational parameters of incinerators in relation to Pak HWM (2005) rules [34] based on WHO guidelines [35]. The Pak HWM rules 2005 specify structure for HWM policy including a waste management team, a waste management plan and weekly record for quantities of generated waste. It is believed that this study will not only evaluate and unveil the differences that lie in management procedures, but also would be helpful to improve the current practices of HWM, which in turn enable the concerned authorities to set directions and implement strategies under the WHO guidelines and appropriate regulatory enforcement.

## Methods

### Study area

The present study has been conducted in Peshawar, Khyber Pakhtunkhwa. Peshawar is located between 33° 44′ N to 34° 15′ N latitudes and 71° 22′ E to 71° 42′ E longitudes. The concerned area cover is of 1257 sq.km and has been considered a historical city due to its geostrategic and socio-economic significance. According to 2017 population census, Peshawar possesses a total population 4,269,079 along with 3.99% average annual population growth rate.

### Study design

The present research is a descriptive, cross-sectional study on HWM in selected public and private hospitals in Peshawar. A comprehensive study was conducted from February to March, 2019 in three government tertiary care hospitals, three government non-tertiary hospitals and ten private teaching hospitals in Peshawar. Only teaching hospitals from public and private sectors with bed capacities of more than 250 beds were selected (Table 1). All the surveyed hospitals are mostly located in residential areas with an average distance of 3.3 km from each other, approximately.

**Table 1:** Characteristics of surveyed hospitals in Peshawar district

Variables (Mean ± SD)	Govt. tertiary care hospitals (*n* = 3)	Govt. non tertiary care hospitals (*n* = 3)	Private teaching hospitals (*n* = 10)
Bed capacity	1393 ± 269	260 ± 15	265 ± 8.3
Estimated No. of daily patients	2600 ± 173	190 ± 36	260 ± 32
Estimated admissions per day	283 ± 76	83 ± 35	60 ± 13

### Data collection and analysis

For this research study, data were collected from the mentioned hospitals after getting the necessary approval from the concerned authorities i.e. Environmental Protection Agency (EPA) and Water and Sanitation Services Peshawar (WSSP) and ethical approval from Institutional Medical Ethics Committee. Data were collected through pretested structured questionnaire based on recommendations by the WHO for evaluation of HWM in developing countries [36]. It consisted of four parts, general information, waste management practices, presence and functional parameters of the incinerator and final disposal of the incinerator bottom ash. Site observations through checklist for the reliability of given information were also included in the survey. The questionnaire was filled during site visits, using information from personnel who were directly related to medical waste management (e.g. administrative officer, facility manager, waste collectors, incinerator operator, engineer and environmental health officer). Questionnaire attached as supplementary material in Additional file 1. The collected data were analysed through SPSS software 25, for the descriptive statistics as well as Kruskal-wallis test for computing statistically significant difference with 95% Cl among public and private sector hospitals for waste generation and fisher’s exact test for hospital waste management practices.

## Results

Initially, hospital waste generation per day and management plan were evaluated. All teaching hospitals have records of the waste generated from their respective institutions. Overall (87.5%), hospitals have plans a hospital waste management committee, sanitary staff and clearly defined procedures for the collection and handling of wastes from specified units (Table 2). Training for HWM team was provided in hospitals (62.5%), while records about trainings was found almost negligible in all surveyed hospitals (Table 2).

**Table 2:** Hospital waste management documented plan and team

Variables	Govt. tertiary hospitals % (***n*** = 3)	Govt. non tertiary hospitals % (***n*** = 3)	Private teaching hospitals % (***n*** = 10)	Overall Hospitals % (***n*** = 16)	Fisher’s Exact test	***p***-value (< 0.05)
**HWM Plan**						
Yes	100	66.67	80	87.5	0.985	1
No	0	33.33	20	12.5		
**HWM team**						
Yes	100	100	80	87.5	0.985	1
No	0	0	20	12.5		
**HWM team training**						
Planned/Done	66.67	0	80	62.5	5.66	0.71
Not done	33.33	100	20	37.5		
**Record of training/workshops files**						
Present	33.3	0	10	12.5	1.796	0.625
Not present	66.7	100	90	87.5		

Furthermore, no recorded data for the quantity of waste generated per bed and its composition per day, both at institutional level as well as in total, were found. General wastes were found to be mixed with health care or infectious waste in all teaching hospitals.

**Fig. 1:**
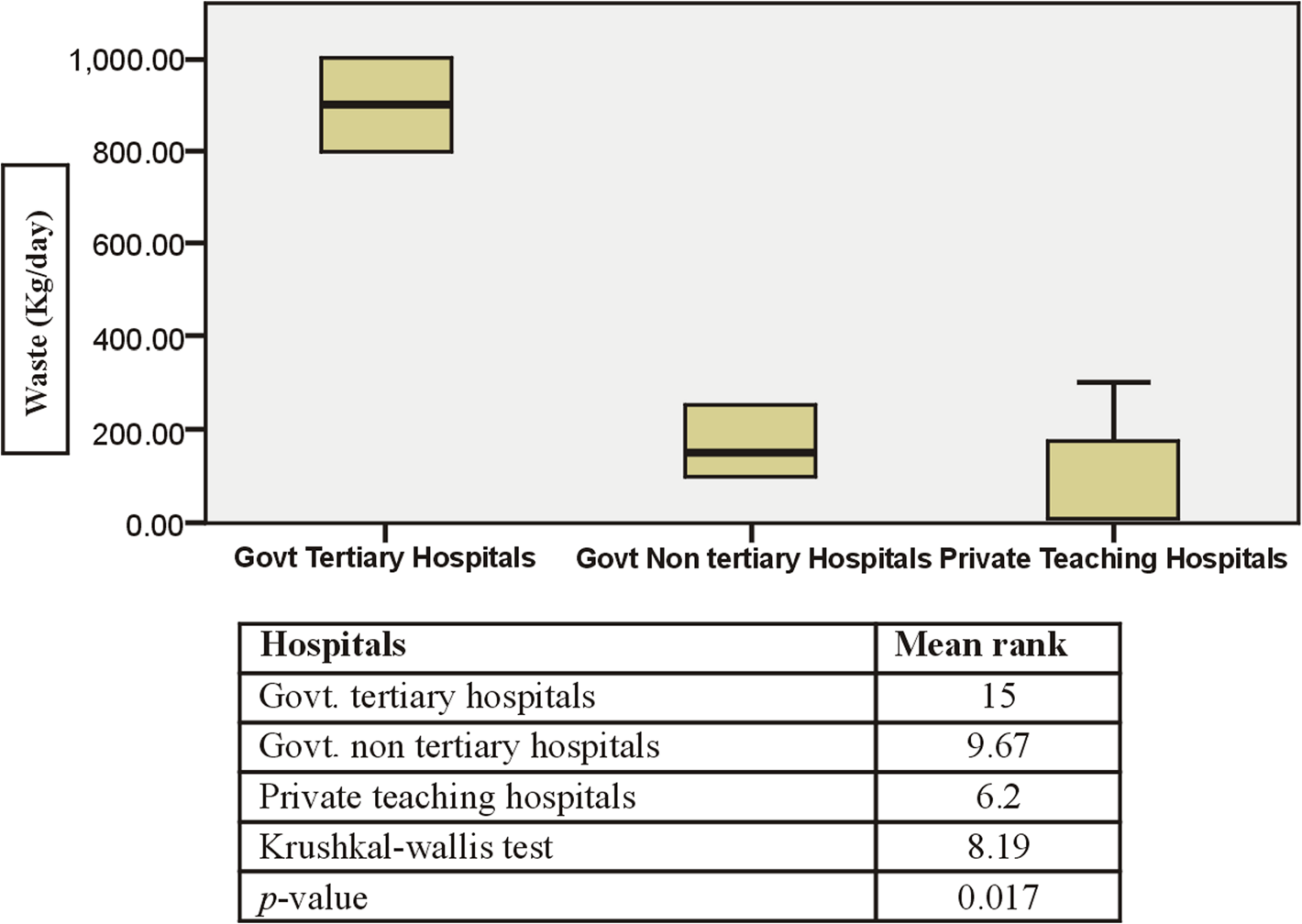
Waste generation in kg/day in surveyed teaching hospitals of the study area

Government tertiary care hospitals produced more waste approximately on average of (900 kg/day) with mean rank of (15), government non-teaching hospitals (166.7 kg/day) with mean rank of (9.67) and private teaching hospitals produced (78.6 kg/day) with mean rank of (6.20) without any segregation at generation point (Fig. 1).

Overall, when the mean ranks of public hospitals were compared with those of private hospitals, significant increased (*p* < 0.017) in waste generation were found in public hospitals due to their more than 1200 patient bed capacity and higher outdoor patient flow and visitors. The summary statistics for pair wise comparison is presented in (Table 3) in which government tertiary hospitals waste generation rate was found statistically different across the private teaching hospitals (*p* < 0.014).

**Table 3:** Krushkal-wallis pair wise comparison for waste generation in surveyed teaching hospitals of the study area

Groups Comparison	Test statistics	Std. Error	***P***-value
Private teaching hospitals-Govt. non tertiary hospitals	3.46	3.11	0.799
Private teaching hospitals- Govt. tertiary hospitals	8.8	3.11	0.014
Govt. tertiary hospitals- Govt. non tertiary hospitals	5.33	3.86	0.504

Current practices of HWM were observed during the site visits and verified through checklist (Tables 4 and 5). It was found that segregation of waste at the generation point was not properly followed in all public teaching hospitals due to patient overload, while only (60%) of private teaching hospitals comply as per WHO guidelines. Hospital staff was not fully aware of proper segregation at the point of generation and collection. Overall percentage was found (37.5%) with no statistical difference between public and private teaching hospitals (*p* > 0.098). The study further states that there is no proper dedicated staff for the segregation of waste. However, the percentage of identified staff was found more (68.75%) as compared to dedicated staff (31.25%) in all of the surveyed hospitals.

**Table 4:** Hospital waste management practices in all surveyed hospitals

Variables	Govt. tertiary hospitals % (***n*** = 3)	Govt. non tertiary hospitals %(***n*** = 3)	Private teaching hospitals %(***n*** = 10)	Overall Hospitals % (***n*** = 16)	Fisher’s Exact test	***p***-value (< 0.05)
**Waste segregation at wards, OTs, labor room etc**						
Yes	0	0	60	37.5	4.781	0.098
No	100	100	40	62.5		
**Dedicated staff**						
Yes	66.67	0	20	25	3.148	0.266
No (Identified staff within the sanitary)	33.3	100	80	75		
**Presence of color coded labeled containers at the waste generation point**						
Yes	100	100	60	75	2.264	0.382
No	0	0	40	25		
**Waste collection method and time**						
Plastic bag/Bin filled	33.3	100	50	56.25	2.835	0.377
Shift end	66.7	0	50	43.75		
**Required facility available for waste transportation (wards, OTs, Labor room) to Storage point**						
Trolleys/ wheel barrowers	0	0	50	50	3.378	0.114
Carrying bags by hand	100	100	50	50		
**Temporary storage area for waste**						
Available	0	0	50	31.25	3.378	0.114
Not available	100	100	50	68.75		
**Presence of color coded labeled containers at the temporary storage point**						
Yes	0	0	50	31.25	3.378	0.114
No	100	100	50	68.75		
**Use of PPE**						
Plastic gloves	100	100	100	100	–	–
Face mask	0	0	0	0		
Apron	0	0	0	0		
Protective shoes	0	0	0	0		
Shades	0	0	0	0		
**Immunization**						
vaccinated	0	0	0	0		
Not vaccinated	100	100	100		–	–
**Availability of incinerator**						
Present (Functional =9)	100	0	60	56.25	5.397	0.72
Not present	0	100	40	43.75

**Table 5:** Operational parameters of the incinerator and final disposal of ash

Variables	Govt. tertiary hospitals % (***n*** = 3)	Private teaching hospitals %(***n*** = 6)	Overall hospitals % (***n*** = 9)	Fisher’s Exact test	***p***-value (< 0.05)
**Type of incinerator**					
Single chamber	0	50	33.33	4.44	0.143
Double chamber	33.33	50	44.45		
Multi-chamber	66.67	0	22.22		
**Temperature range (800-1200 °C) is achieved in both chambers before waste introduced**					
Upto range	33.33	50	66.67	0	1
Not upto range	66.67	50	33.33		
**Chimney height (> 4 m)**					
Up to standard	33.33	50	66.67	0	1
Not up to standard	66.67	50	33.33		
**Incineration operation manual**					
Available	66.67	66.67	66.67	0	1
Not available	33.33	3.33	33.33		
**Incinerated waste daily/monthly record**					
Available	0	0	0		
Not available	100	100	100		
**Type of arrangement available for final disposal of ash**					
Container	100	83.33	88.89	0	1
Cemented pits	0	16.67	11.11		

Waste collecting bins were observed in all public sector hospitals (100%) and in private teaching hospitals (60%) with proper labels and color codes. All the government tertiary care hospitals were not maintaining appropriate shifting of wastes from smaller bins to larger containers at disposal point. Most often, in both sectors, the waste was being collected in shopping bags/bins once filled (56.3%) carried out by waste handler (50%) to the temporary storage point. As such no proper availability of trolleys and wheel barrowers for waste transportation were observed in public hospitals (0%). Statistically, no significant difference was observed in both public and private sector hospitals (*p* > 0.05) in terms of required facilities for waste collection from generation point to temporary storage area.

However, about (50%) in private teaching hospitals, waste collection was done by means of trolleys or wheel barrowers for on-site transportation of hospital waste and is not used for other purpose. Site survey also revealed that only (50%) private teaching hospitals have temporary storage area within the hospital premises with color coded labeled containers (50%) for waste segregation, whereas rest of the hospitals dumped waste in open area. Staff in the hospitals was handling the waste without using proper protection measures except gloves (100%) and were not aware of the potential hazards. In all surveyed hospitals, none of the observed waste handler and staff wore face masks, aprons or protective shoes. No record for vaccination against hepatitis A & B and Tetanus were found for the protection of workers who are in daily contact with waste handling and collection (Table 4).

Overall, (56.3%) of the hospitals had been equipped with incinerator, out of which (100%) in government tertiary care hospitals, no incinerator in govt. non tertiary hospitals and (60%) in private teaching hospitals. Observed incinerators were locally made and found not environmentally friendly as they used old technology and operations were not up to the minimum standards like temperature range and chimney height etc., as shown in the (Table 4). Approximately (33.33%) incinerators were found single chamber, (44.4%) double chamber and (22.22%) multi-chamber in private and public sector hospitals respectively.

Coal and sometimes diesel/kerosene are used as fuel in the incinerator due to shortage of natural gas, which is a potential source of toxic air pollutants. Availability of an incinerator operational manual was also checked and found (66.67%) in hospitals (Table 5).

Those hospitals which have no incinerator facility (43.75%) were mostly practicing open dumping/burning of the waste in the vicinities. They have 3 or 5 years contracts with private companies other than WSSP for ash burial/open dumping of waste in their own lands or fields without realizing the hazardous effects of the waste on health and environment.

Ash generated at the incinerator was not being buried deeply in the 4 or 5 ft cemented pits/trench. Only (11.11%) hospitals including (16.67%) private teaching hospitals have cemented pits arrangement for final disposal of ash, while (88.89%) hospitals used container including (100%) government tertiary care hospital and (83.33%) private teaching hospitals. No documentation record for incinerated waste was found in all hospitals. In the present study, as such, no HWM practices were observed for Government non-tertiary hospitals.

## Discussion

Comparing the present results with other studies around the world, as well as some studies conducted in major cities of Pakistan, clearly indicates that there was no proper, systematic management of hospital waste practiced [24, 29, 37–42]. Although, (87.5%) surveyed hospitals have HWM plan and team with specified responsibilities. In addition to this study, the study of Harhay et al., [43] showed that six countries including China, India, Brazil, Pakistan, Bangladesh and Nigeria, the top ten most populous countries in the world, were found to be facing inadequate HWM problems.

These poor management practices are not only due to the lack of interest from the hospital management team or lack of awareness concerning health risks, but also due to the economic issues in implementation of healthcare policy from the government [8].

Although 20 years ago, WHO issued documents assessing in improving the waste management from hospitals but unfortunately did not trigger any change in Pakistan.

The study of Zeeshan et al., [42] about HWM polices in Pakistan revealed partial presence of HWM plans, poor record keeping of waste produced and lack of dedicated budget for HWM. In this context, Pak HWM- 2005 rules still need positive improvement and additional provision to become in alignment with the WHO standard guidelines [37]. The better hospital waste management can be perceived through effective legislation of healthcare waste management and can be witnessed in Kingdom of Bahrain; where proper management of the healthcare waste practices showed positive signs of improvement due to amendments and revisions for improvement in national healthcare waste management legislation [44].

In the present study, as such no significant difference were found in hospital waste management practices except waste generation per day which was found comparatively higher in tertiary care hospitals (*p* < 0.017) than private teaching hospitals. This may be attributed due to high in patient flow, provision of services and greater bed capacity, but required more attention and efforts to train the hospital waste management team so as to prevent the infections stemming from the waste.

However, there was found no documented/maintained record of waste by type at the point of generation per bed in the studied hospitals.

Usually, private teaching hospitals were witnessed better than the public ones in some fields of waste management. Findings of the present study are similar to a study conducted in Islamabad which revealed that the practices of hospital wastes are better in private hospital than the public one [45]. Similarly, in other study conducted by Khan et al., [40] in four tertiary care hospitals of Peshawar, i.e. two hospitals from public and two from private sectors revealed that private hospitals performed well practices for waste management as compared to public hospitals. Although private hospitals may charge more and hence have less patients but may be more inclined to follow better HWM practices in comparison to public hospitals where the conditions are just reversed. The most prominent reasons for relatively better practice of waste management in the private teaching hospitals are waste segregation, storage, training and awareness and staff availability etc., but are unable to fully implement and practice the HWM rules- 2005.

Generally, the segregation of waste at the generation source is considered as one of the crucial components for efficient HWM practices but unfortunately, it is not followed properly as per WHO guidelines which recommend that “hospital waste be separated in distinct groups with regard to the requirements of disposal and treatment”. Improper segregation could convert rest of the general waste into hazardous waste and poses a potential threat to all the stakeholders including healthcare providers, patients, visitors and surrounding communities [46]. Studies from other developing countries were also in lined, that there was found no proper segregation of waste into different groups at generation point for proper disposal. Mostly, hospital waste collected from different units was dumped along with general waste for further disposal [10, 47]. Likewise, (75%) surveyed hospitals had color code and labeled containers but in fact, there was seemed no proper supervision from HWM team/administration for waste segregation at the generation point. Apart from this, (31.25%) of studied hospitals used the color code and labeled containers at temporary storage area. About (68.75%) of surveyed hospitals had no temporary storage area for waste and practiced open dumping. As per HWM [34] rules, waste needs to be collected at least once daily in accordance with the schedule specified in waste management plan. The removed waste bags and containers need to be replaced with new ones of same type, but unfortunately the present conditions are most horrible. Mahwish et al., [48] in their study revealed the same conditions in both public and private hospitals of Islamabad, Karachi, Lahore, and Khyber Pakhtunkhwa of Pakistan.

In (50%) hospitals, on site transportation of waste was mostly done manually, using plastic bags, while off- site transportation was undertaken with the use of trucks, by had contracts with different government and private authorities. Highlighting the hazardous nature and involving high risks of waste handler in terms of getting injury or contact with disease causing pathogen, Johnson et al., [49] in their study described that off-site transportation of hospital waste on roads must be carried out by trained staff in a dedicated vehicles with closed containers. Similarly, Patil et al., [50] in their study illustrate that “management of hospital care waste depends on the input from the administration and active participation by trained staff in segregation, storage, collection, transportation, treatment and disposal”.

Furthermore, in surveyed hospitals, the identified staff/waste handler as well as HWM staff did not use all the required PPE (i.e. plastic gloves, face mask, apron, protective shoes and shades) except wearing gloves; such staff handled and transported the waste without realizing the high risks in case of injury and accidently being in contact with disease causing pathogens [19]. A study conducted by [51] in Karachi among health care workers reported high prevalence of hepatitis B infection, among 20% sweepers of a medical center due to unsafe disposal of hospital waste. Similar results have been reported in various studies, highlighting the importance of PPE for waste handlers, while dealing with potentially dangerous waste particularly sharps, blood and blood contaminated fluids [52–54]. Also no maintained record of vaccination for protection against from hepatitis A & B and Tetanus were found for HWM team.

Currently, three kinds of methods are being used for disposal of waste, i.e. incineration, landfills, and open dumping. Neither a single landfill is constructed on scientific lines nor do the installed incinerators at various places have proper structure and operational parameters. The most prevalent type of waste treatment was observed as incineration and open burning and finally the waste disposed together with general waste in the open disposal site. Besides, in the studied hospitals, 9 out of 16 hospitals have incineration facility for final disposal of waste in which 5 private teaching hospitals uses single chamber incinerator built of brick. Several problems have been reported with single chamber brick-made incinerators, including emission of toxic substances (SOx, NOx, HC1, smoke, furans and dioxin gases) into the environment that are a risk to public health [10, 19, 21]. Moreover, partial and incomplete burning in locally made incinerator increase the risks of hazards by contaminating the land and water resources on disposal [55]. They are mostly situated in densely populated areas with an average distance of 3.3 km. Though in WHO [56] guidelines, it is clearly mentioned that off-site treatment can be more easily ensured in one centralized facility than in several plants.

The rest of the hospitals practicing open dumping of waste or have did 3 or 5 years contracts with government and some private authorities for off-site waste transportation and dumping in their own lands without realizing the deteriorating effects on the environment as well as residents in the surrounding. This indicates a void in implementation of the HWM [34] rules for the adequate management and treatment of hospital waste.

## Conclusion and recommendations

This study was conducted to evaluate the HWM practices in teaching hospitals of Peshawar in connection with implementation of WHO recommended guidelines. The overall findings of the present study indicated, lack of HWM practices in all surveyed hospitals. However, waste generation capacity of public sector hospitals was found significantly high (*p* < 0.017) as compared to private teaching hospitals. In all studied hospitals, only nine hospitals have had their functional incinerator for hospitals waste combustion, while the rest of the hospitals were practicing open dumping of waste or off-site transportation. Functional incinerators were locally built, not up to the standard as recommended by WHO, and located in densely populated areas of Peshawar. Similarly, awareness regarding proper waste management practices remains low in the absence of training for hospital staff. Further, waste handler operates without the provision of safety equipment or immunization. It is concluded that HWM across the Peshawar faces several challenges and require sustainable waste management practices on long way in reducing the harmful effects of hospital wastes both at institutional as well as at community level. Therefore this study offers the following recommendations;

- Basic training and capacity building program for HWM staff with regard to use of PPE, maintain daily record of waste generation/bed and ensure proper segregation to final disposal of waste should be arranged on regular basis by concerned authorities along with the provision of reinforcement training material.
- The Government concerned authorities should create /develop inspection teams for regular monitoring and continuous supervision of HWM staff and effective implementation of HWM practices in hospitals.
- The inspection team also strictly enforced the national HWM 2005 rules in all hospitals and penalties to be imposed in case of contravention.
- Open dumping of waste should be avoided and a specific place should be declared and designed as temporary storage from where proper transportation and disposal of waste be ensured.
- A common hospital waste treatment facility/a centralized incinerator equipped with new technologies should be installed in outside of the / away from the residential area which can efficiently cater all the HWM needs of the hospitals. This facility will not only minimize the risks of deteriorating the air quality of the residential areas and ill effects but also will be helpful to save the cost associated with waste disposal via incinerator.

## Supplementary Information

**Additional file 1.**

## Data Availability

The authors confirm that the summary of data supporting the findings of this study is available within the article. However, detailed data of this study are available from the corresponding author upon request.
