# Supplementary material for: Current practices of waste management in teaching hospitals and presence of incinerators in densely populated areas
Source: BMC Public Health. 2021 Jul 7;21:1340. doi: 10.1186/s12889-021-11389-1 (PMC8262056; doi:10.1186/s12889-021-11389-1)
Supplement: Supplementary file 1 — Additional file 1. [file 12889_2021_11389_MOESM1_ESM.docx]

**Appendix-I**

**This questionnaire is purely for research purpose**

**Current practices of waste management in teaching hospitals and presence of incinerators in densely populated areas**

**Questionnaire**

**Part-1: General information**

1. Name of the Hospital: ___________________________________________
2. Date of visit:___________________________________________________
3. Prescribed level of health facility? (A) Govt. tertiary hospital

(B) Govt. non tertiary hospital

(C) Private teaching hospital

1. Person interviewed? (A) Administrative office

(B) Facility manager

(C) Waste collector

(D) Incinerator operator

(E) Engineer

(F)Environmental health officer

1. How long have you worked in this hospital? (A) 1 year

(B) Less than 5 years

(C) More than 5 years

**Part- 2: Waste management practices**

1. Is hospital waste management plan exist? (A) Yes (B) No
2. Is formal waste management team/committee present? (A) Yes (B) No
3. Is there any training workshop done/planned for hospital waste management team?

(A) Yes (B) No

1. Is training files/record available? (A) Yes (B) No
2. How much hospital waste is generated (Kg) per day/daily?
3. What fraction of the waste is infectious? (A) 0-15%

(B) 15-30%

(C) More than 30%

(D) No record available

1. Is there present any documented record of generated waste? (A) Yes (B) No
2. Is waste segregated at wards, OTs labor rooms etc (A) Yes (B) No
3. Is dedicated staff available for hospital waste management? (A) Yes (B) No
4. If not, have the personnel within sanitary staff been identified/selected for this purpose? (A) Yes (B) No
5. Are color coded containers present at the waste generation point? (A) Yes (B) No
6. How often the containers emptied?/What are the waste collection method and timings? (A )Once a day

(B) At the end of the shift

(C) When Bag/bins filled

1. Is required facility available for waste transportation (wards, OTs, Labor room) to storage point? (A) Trolleys/wheel barrowers

(B) Carrying bags by hand

1. Is temporary storage area available for waste disposal? (A) Yes (B) No
2. Are color coded labelled containers present at the temporary storage point?

(A) Yes (B) No

1. Do you use PPE for protection (Plastic gloves, face mask, apron, protective shoes, and shades)? (A) Yes (B) No
2. How often you are immunized against hepatitis A & B and Tetanus?

(A) Once a year

(B) No idea

(C) Not vaccinated

**Part- 3: Presence and functional parameters of the incinerator**

1. What type of incinerator do you have? (A) Single chambered

(B) Double chambered

(C) Multi chambered

1. What fuel type is used for ignition? (A) Diesel/Kerosene

(B) Gas

(C) Coal

1. Is Incinerator functional or in service? (A)Yes (B)No
2. How is the combustion temperature measured?
3. Is 850^o^C temperature achieved in both chambers before waste introduced?

(A)Yes (B) No

1. Is 1100^o^C temperature achieved in second chamber prior to introduction of such waste? (A)Yes (B) No
2. Is it per standard to have the incinerator chimney height > 4m?

(A)Yes (B) No

1. Is incineration operation manual available? (A) Yes (B) No
2. Is incinerator daily record available? (A)Yes (B) No
3. Is incinerator monthly record available? (A)Yes (B) No
4. Is incinerator maintenance and repair log available? (A)Yes (B) No
5. Is the incinerator operator understand the operational procedures?

(A)Yes (B) No

1. Is the incinerator operator understand or follow the safety procedures?

(A)Yes (B) No

**Part-4: Final disposal of the incinerator bottom ash**

1. Is there present any arrangement for the treatment and final disposal of incinerator bottom ash? (A)Yes (B) No
2. If yes, what type of arrangement present? (A) Cemented pits

(B) Container

(C) Contract
